# Contextualizing a framework for improving postnatal care in Ethiopia

**DOI:** 10.3389/fpubh.2022.919175

**Published:** 2022-08-23

**Authors:** Elias Teferi Bala, Lizeth Roets

**Affiliations:** Department of Health Studies, University of South Africa (UNISA), Pretoria, South Africa

**Keywords:** contextualizing a framework, postnatal care, improving postnatal care, maternal health, quality postnatal care

## Abstract

**Background:**

Postnatal care is among the major recommended interventions to reduce maternal deaths. To improve the low postnatal care utilization in Ethiopia, the framework developed for this purpose in Kenya was contextualized and adapted for implementation in the Ethiopian context.

**Objectives:**

The objectives of this article are to share the process followed to contextualize Chelagat's framework for improving postnatal care, for the implementation in Ethiopia as well as the finalized contextualized framework.

**Methods:**

A quantitative descriptive research design was adapted. A self-administered questionnaire was used to gather data during November 2018 from 422 postnatal care providers and coordinators, using stratified random sampling. The AGREE II was utilized to assess adaptability and applicability and an open-ended question allowed to assess the challenges and opportunities for utilizing the framework. The data were analyzed using SPSS computer software, Version 23.

**Results:**

The findings revealed that the framework from Chelagat was adaptable to use for the improvement of postnatal care in the Ethiopian context. The results from the analysis of the data using AGREE II indicated an average domain score of 92%, for contextualization possibility.

**Conclusion:**

The framework originally developed by Chelagat was contextualized and refined to be implemented in Ethiopia to improve postnatal care.

## Background

Despite the goals set by the United Nations (UN) to reduce maternal mortality to <70 per 100,000 live births, the rates in Sub-Saharan Africa are unacceptably high (1, 2, 40). The maternal mortality rate in Ethiopia remained stagnant over the past years as it averaged between 412 and 871 per 100,000 live births (1). Most maternal deaths occur during the first 48 h after delivery, despite this time, being critical for monitoring the complications arising from the delivery (3) and thus preventing the maternal mortality.

More or less 65% of maternal deaths occur within 1 week of delivery and nearly 80% of maternal deaths occur within 2 weeks of delivery, (6, 7) thus during the most critical first few days after birth (3, 8). The vast majority of maternal deaths, within the first 48 h after delivery are preventable, providing the delivery of optimal postnatal care (4, 5). The maternal morbidity is associated with missed opportunities for preventive measures such as early diagnosis and treatment (8).

Timely quality postnatal care during the first hours and days after birth is essential for improving maternal and newborn health (8–10). Despite accessible postnatal care being positively associated with improve maternal and neonatal mortality and morbidity rates (11, 12), only 17% of women in Ethiopia receive postnatal care during this crucial time (3).

Despite the many advantages of postnatal care, it remains a neglected part of maternal and child health service in most developing countries (13, 14) including Ethiopia. Most mothers do not receive postnatal services from competent health professionals (15, 16).

Quality postnatal care should be a comprehensive or integrated package that covers a range of aspects that affect both the mother and baby. Postnatal services should (1) provide the support for the mother and her family in the transition to a new family, (2) contribute to the prevention of health problems in mother and baby, (3) ensure the early diagnosis of possible complications, (4) provide the treatment for complications that occur in mothers and infants, (5) refer the mother or infant for specialist care when necessary, (6) provide health education on baby care, maternal nutrition, and contraception, (7) support and promote breastfeeding, (8) provide contraception service, and (9) provide an immunization service for the infant (8, 17).

Kenya and Ethiopia are Sub-Saharan countries experiencing similar challenges pertaining to postnatal care. In Sub-Saharan countries including Kenya and Ethiopia, the inacessblity of health services, misconception about postnatal care as well as diverse culture and norms are some of the challenges contributing to low postnatal care utilization (19, 20). To assess the possibility for adapting or contextualizing a framework developed for the Kenyan context in Ethiopia, broad similarities and differences were worth taking note of.

### Postnatal care utilization

Postnatal care coverage in Ethiopia is extremely low with only 19% women receiving care, thus 81% of women not receiving postnatal care (3). Furthermore, 95% of those who did receive care, did not receive care within 2 days after delivery as is recommended (3, 8). In Kenya, 53% of women receive postnatal care (18), which is more than double compared with that of Ethiopian women. As both countries share similar socio–demographic and socioeconomic characteristics, similar challenges pertaining to accessing and utilizing postnatal care are experienced; amongst others are (1) the recognized 40-day period of rest and staying indoors (19, 20), (2) cultural misconceptions of postnatal care, (3) lack of awareness of the advantages of postnatal care, (4) limited financial resources and transport costs, (5) accessibility challenges, and (6) the long distances to health facilities (21–23).

Challenging to postnatal care delivery in both countries is the reported negative attitudes of health care providers (24, 25), hindering postnatal care utilization. Despite the efforts made in Ethiopia to decrease the maternal mortality by increasing maternal health care utilization (including postnatal care) through different strategies, such as health sector reforms, increasing the number of trained health professionals, and health facilities, there was neither a significant reduction in maternal mortality nor an increase in postnatal care utilization (3).

### Chelagat's framework

In 2015, Chelagat developed a framework that was validated and recommended for implementation in Kenya, as well as in other countries with similar context, for improving postnatal care. Implementation of this framework can ultimately improve maternal and neonatal outcomes in countries with similar challenges.

**Figure 1:**
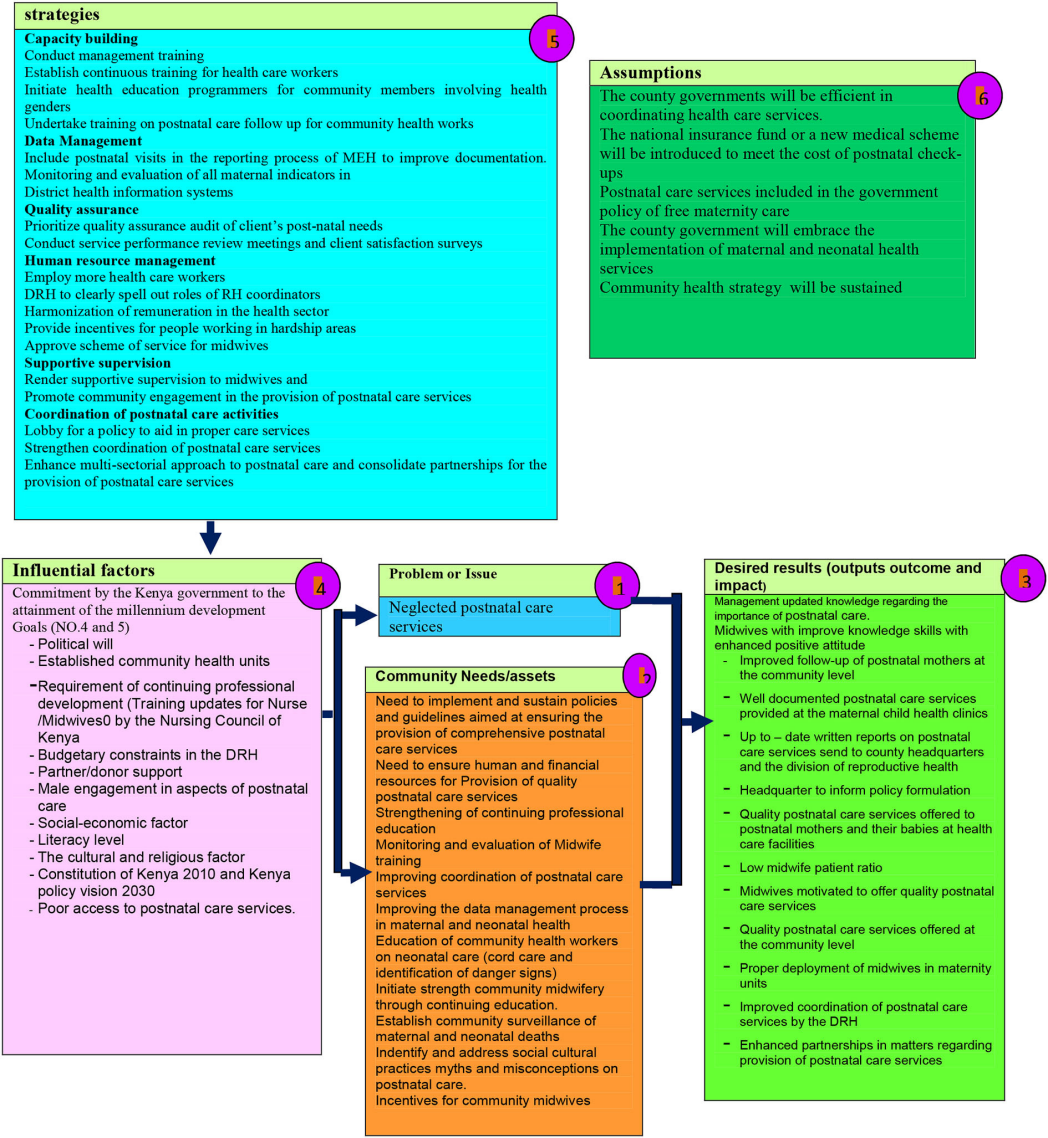
Framework for improving postnatal care utilization [Chelagat (33)].

Chelagat's framework, underpinned by the Systems Model, was developed and guided by the Theory of Change Logic Model (Figure 1). This framework presents a picture of how a certain effort or initiative to improve postnatal care is supposed to work. It explains why the strategies identified in the framework are a good solution to the experienced challenge (26). The framework is a systematic and visual way of understanding the relationship among the resources required to operate postnatal care services, the activities to be undertaken, and the results that the framework hopes to achieve (27).

In Chelagat's framework, neglected postnatal care in Kenya was identified as the main problem to be addressed (Figure 1, number 1). This problem is in accordance with the problem identified in Ethiopia (Figure 1). The assumptions for implementing the framework (Figure 1, number 6) relate to the expectations or predictions behind how certain factors could affect strategies addressing neglected postnatal care. The identified strategies to improve postnatal care in Kenya, as indicated in the framework (Figure 1, number 5), were expected to be similar to those that might improve postnatal care in Ethiopia. The framework also includes the intended results or outcomes (Figure 1, number 3) and the influential factors (Figure 1, number 4) for both problem and implementation of the framework (26–28).

## Methods

### Study design

A quantitative descriptive cross-sectional study design was employed to assess, adapt, and contextualize the framework developed for the Kenyan context for the possible implementation in Ethiopia.

### Population

The study was conducted in Oromia regional state, purposively selected as it is geographically large in comparison with other regions and nearly one-third of the Ethiopian population live in the Oromia region (3).

The stakeholders (national and provincial reproductive health coordinators as well as all midwives and nurses allocated to midwifery units in all hospitals in Kenya) participating and contributing to the development of Chelagat's framework, were similar to the stakeholders involved in postnatal care in Ethiopia. Therefore, the postnatal care service providers, namely, midwives, nurses, and health officers at health facilities, as well as the district, regional, and national reproductive health coordinators in the Ethiopian health system assisted in adapting and contextualizing the framework.

### Sample size and sampling procedure

The postnatal care providers and coordinators working at the 80 health centers, 25 hospitals, the district and regional postnatal care coordinators in the district health departments, as well as the regional health department, formed the population. The identified populations are key to postnatal care in the Ethiopian health system. The total number of participants comprised of 422 participants (294 nurses, 74 midwives, 46 health officers, 6 district postnatal care coordinators, and 2 regional postnatal care coordinators). The study respondents were selected by using stratified simple random sampling as there was a sampling frame prepared by the researcher based on the existing lists of postnatal care providers and coordinators at each health facility or health department.

The total sample size was determined, using the single population proportion formula as described by Kothari (29):


$$\begin{array}{rcl} n\; & = & \;Z\left(\alpha /2\right)2^{*}P\;\left(1\;-\;P\right)/d2\\ n\; & = & \;{{\left(1.96\right)}^{2}}^{*}{1\left(1\;-\;0.5\right)/\left(0.05\right)}^{2}\\ n\; & = & \;384 \end{array}$$

The assumptions under this formula were as follows:


$$\begin{array}{l} \;n\;=\text{ sample size};\;\\ Z(\alpha /2)=\text{ the value of normal distribution representing a }\\ \text{ confidence level of 95}\%.\text{ Its value is 1}.\text{96};\;\\ \;P\;=\text{ proportion of the case};\\ \;d\;=\text{ margin of error}. \end{array}$$

Considering a non-response rate of 10%, the final sample size was 422.

The sampling frames were the lists of individuals from which the researcher selected the sample, namely, the lists of postnatal care providers and postnatal care coordinators.

### Research instrument

A questionnaire was used to collect data from each participant to assess applicability, for the adaptation and contextualization of the framework in the Ethiopian context. Ten trained field workers were purposively selected from university graduate nurses to gather the data. Before commencement of the actual data collection, a pilot study was conducted with 5% (21) of the participants who were outside the study area but who were postnatal care providers and coordinators (30).

The AGREE II, a standardized and tested questionnaire (30, 31), was used for data collection. Additional questions, guided by standardized WHO guidelines, were added to the AGREE II to assess baseline information on postnatal care services in Ethiopia as to compare the data, with that available from Kenya. The data from the questionnaires were analyzed with the assistance of a statistician. The Statistical Package for Social Scientists (SPSS) computer program, Version 21 was used to process and analyze the data. Descriptive statistics, using frequencies and percentages for categorical data were used, and the results for this study were presented in text form, in tables, and in pie charts.

For guideline or framework adaptability assessment, AGREE is a commonly used instrument. The instrument allows for assessing methodological rigor and transparency in which a guideline or framework is developed. The original AGREE instrument has been refined, which has resulted in the new AGREE II version and a new user's manual which was published in 2013 (31). AGREE II includes 23 appraisal criteria (items) organized within six domains and two overall assessments as follows: (1) Overall framework quality; (2) Recommendation for use. The six domains are (1) overall aim of the guideline, (2) stakeholder involvement, (3) rigor of development, (4) clarity of presentation, (5) applicability, and (6) editorial independence.

Each domain in AGREE II assesses, by means of a Likert scale, a unique dimension of the developed framework quality. The participants were allowed to provide individual opinions on what they believe would improve the quality of the guidelines or framework to be contextualized in the space that was provided additional to the AGREE II tool.

### Data gathering process

Ten data collectors (6 males and 4 females) from a university college of medicine and health sciences were purposively selected for assisting with data collection. They were chosen based on their experience as fieldworkers. They shared with all eligible respondents an information and recruitment letter them and provided a consent form to complete, if volunteering to participate. The data collectors distributed the questionnaires to all consenting participants, requesting them to complete it in private and return the completed questionnaire within 2 days.

### Data analysis

The data analysis was done, using statistical procedures as well as open coding for the open-ended questions. Quantitative data were analyzed, summarized, and presented in tables and pie charts using frequencies and percentages (32), applying the principles suggested by AGREE II (2013:10). The analysis of the questionnaire, thus, was based on the following six domains indicated in AGREE II: (1) Scope and Purpose; (2) Stakeholder Involvement; (3) Rigor of Development; (4) Clarity of Presentation; (5) Applicability, and (6) Editorial Independence.

As recommended in AGREE II, each of the AGREE II items and the two global rating items were rated on a 7-point scale that ranged from 1 to 7 (1–strongly disagree to 7–strongly agree). The AGREE II User's Manual section was used to rate each domain. Accordingly, the AGREE II was used to calculate the domain scores by summing up all the scores of the individual items in a domain and scaling the total as a percentage of the maximum possible score for that domain calculated as follows:


$$\begin{array}{l} \text{Maximum possible score = 7 (strongly agree) × number of}\\ \text{ items × 422 (appraisers).}\\ \text{Minimum possible score = 1 (strongly disagree) × number}\\ \text{ of items × 422 (appraisers).} \end{array}$$

As recommended in AGREE II, the scaled domain for the contextualization possibility of the framework was assessed using the formula (31):


$$\begin{array}{l} \text{The scaled domain}:\;\\ \;\frac{\text{Obtained score - Minimum possible score}}{\text{Maximum possible score - Minimum possible score}} \end{array}$$

An inductive thematic analysis was employed for the narrative data from open-ended questions. Themes and categories were identified after the data were read and coded so that similar ideas were grouped as themes that were underpinned by categories.

### Ethical consideration

Ethical approval to conduct the study was obtained from the ethics committee from the custodian university (HSHDC/452/2015). Permission and support to conduct the study and gain access into the field were obtained from the respective administrative offices of Oromia Regional State Health Bureau, each health facility, including health centers, hospitals, district, and regional health departments as well as from each individual respondent who volunteered to participate.

## Results and discussion

### Biographical data (*N* = 422)

All 422 respondents volunteered to participate and completed the questionnaires. Table 1 presents the sociodemographic characteristics of the respondents. Among the respondents, 27.3% were younger than 30 years of age, 0.9% were older than 60 years of age, and 33,6% were between 30–39 years of age (Table 1). This indicated nearly all respondents from all professions, who were within the productive age group that positively influences postnatal care services as supported by Dlamini (9).

**Table 1:** Sociodemographic characteristics of respondents.

**Variables**	**Category**	**Frequency (*F*)**	**Percent (*f*)**
Age	Younger than 30 years	115	27.3
	30–39 years	142	33.6
	40–49 years	122	28.9
	50–59 years	39	9.2
	60 years and older	4	0.9
Gender	Male	174	41.2
	Female	248	58.8
Educational level	Master's	34	8.1
	Bachelor's	263	62.3
	Diploma	125	29.6
Work experience in years	1–10 years	234	55.5
	11–20 years	151	35.8
	21–30 years	37	8.8

A total of 58.8% of the respondents in this study were females and 41.2% were males (Table 1), similar to in Kenya where 88.5% of the postnatal care providers were females (33, 34).

Many respondents were in possession of bachelor's degrees (62.3%) or diplomas (29.6%) in a health care-related profession. The Ethiopian health care system is very fortunate to have more degree-holding nurses than Kenya, where only 1.96% of nurses were degree holders (33). Nurses, midwives, and other health care providers with advanced qualifications have more advanced knowledge and skills to offer standardized and quality postnatal care to mothers and newborns (35, 36), and therefore the Ethiopian health system is privileged. Only a small number (8.1%) were in possession of master's degree in a health care-related profession. This is of concern as those with postgraduate qualifications can conduct research, change policies, and influence decisions made by policy makers (36).

Two hundred and thirty-four (55.5%) of the respondents had work experience of 10 years or less, 35.8% had 11–20 years of experience and 8.8% over 21 years of experience in postnatal care service delivery, thus they have adequate and relevant clinical experience in postnatal care services to contribute to effective and standard postnatal care provision (37). The mean duration of work experiences among postnatal care providers in Ethiopia is similar to that reported in Kenya, where it was 11.22 years (33).

### Adaptation possibility assessment for Chelagat's framework in the Ethiopian context (application of agree II)

The possibility to adapt the Chelagat framework in Ethiopian context was described according to the findings received from each specific domain of the AGREE II tool. The AGREE II tool has six domains on which the possibility of adapting is described.

#### Domain 1: Scope and purpose of the framework

Domain 1 is concerned with (1) the overall aim of determining the framework's possibility to be contextualized, (2) the specific health problem (the neglected postnatal care) to be addressed, and (3) the target population, to whom the framework is meant to apply, is to be assessed. This domain deals with the potential health impact of the contextualized framework on postnatal mothers, families, the community, and the health system.

The assessment was based on whether the overall objective of the framework, namely, the expected improvement in postnatal care as a result of the implementation of the contextualized framework, can be achieved in Ethiopia. It also considered whether the health question, the problem (neglected postnatal care, as identified in Kenya), is relevant within the Ethiopian context as well as whether the framework clearly described the target population to whom the contextualized framework will apply.

Moreover, AGREE II does not stipulate minimum domain scores or patterns of scores across domains to differentiate between high-quality and poor-quality guidelines. However, it recommends that decisions should be made by the user and guided by the context in which AGREE II is being used (31). Therefore, a domain score of 75% or more was considered for all domains to indicate a high-quality framework recommended for use in the Ethiopian context.

For domain 1, the average scaled score of the assessment was 93.4%. With this average score of 93.4%, the participants, therefore, believed that the objectives of the framework and the problem that needs to be solved in Ethiopia were similar to the problem in Kenya (neglected postnatal care), indicating that the implementation of this framework can improve postnatal care within the Ethiopian context (Table 2).

**Table 2:** Domain scores (*n* = 422).

**Domain**	**Profession and number of appraisers**	**Total number of items**	**Minimum possible score**	**Maximum possible score**	**Obtained score**	**The scaled domain score**
The scope and purpose	422	3	1,266	8,862	8,864	93,4
Stakeholder involvement	422	7	2,954	20,678	18,899	90
Rigor of development	422	10	4,220	29,540	27,605	92
Clarity of the framework	422	3	1,266	8,862	8,339	93
Applicability of the framework	422	4	1,688	11,816	11,120	93
Editorial independency	422	1	422	2,954	2,769	94
Implementation of the framework will improve the provision of postnatal care in the Ethiopian context	422	1	422	2,954	2,748	92

#### Domain 2: Stakeholder involvement

The second domain focused on the extent to which Chelagat's framework was developed by the appropriate and similar stakeholders to that of Ethiopia and whether it addressed the views of all the intended users. The assessment was made using seven items, namely, (1) whether the relevant group of experts participated in the development of the framework, (2) whether the views and preferences of the target population were taken into consideration, (3) if the factors that affect postnatal care utilization were included, (4) if the strategies for improving postnatal care were included, (5) whether the role of the community in improving postnatal care was considered, (6) if the outcome on postnatal care, if implemented, were indicated, and (7) whether the target users of the framework were clearly defined.

The average scaled score of the assessment was 90% (Table 2). The participants, with an average score of 90%, found the framework to have been clearly described and the relevant group of experts participated in the development. This finding is an indication that the implementation of this framework could therefore be adapted for the Ethiopian context.

#### Domain 3: Rigor of development

Domain 3 assessed issues related to the process used to gather and synthesize the evidence, the methods to formulate the recommendations, and updating them. The specific assessment was made on whether the developed framework (1) used systematic methods, (2) followed clear criteria for selecting evidence, (3) described the strengths and limitations of the body of evidence clearly, (4) described the methods used for formulating the recommendations clearly, (5) considered the health benefits, (6) considered the health side effects in making the recommendations, (7) considered the health risks in formulating the framework, (8) considered the link between the recommendations and the supporting evidence, (9) explicitly involved postnatal care experts for improvements of postnatal care, and (10) provided a procedure for updating the guideline.

An average scaled score of the assessment of domain 3, was 92% (Table 2), thus indicating that the framework was developed according to all 10 aspects listed above. This result indicated that the scientific rigor of the framework developed by Chelagat was of high quality which, in turn, indicated that all aspects were included during the development and therefore relevant for implementation in the Ethiopian context.

#### Domain 4: Clarity of the framework

Domain 4 is concerned with the language, structure, and format of the developed Chelagat framework. When a framework is developed for improvement in a health-related field, the framework developers expect that it should provide a concrete and precise description of—in this study's context—the strategy, how the strategy will be implemented, and what segment of the population should be involved, as informed by the body of evidence. It is important to note that, in some instances, the evidence may not always be clear and there may be uncertainty about the best care option(s) or strategies.

When such doubts exist, the framework developers are expected to make evidence clear to enhance the implementation of the framework (31). For the implementation of the framework, postnatal care providers and coordinators should be able to easily find the most relevant recommendations and evidence in the framework. The clarity of the framework developed by Chelagat to improve postnatal care in Kenya was assessed in this study focusing on whether the framework (1) is specific and unambiguous, (2) the different options for the provision of postnatal care are presented in the recommendations, and (3) used key recommendations that are easily identifiable.

The average scaled score of the assessment of domain 4 was 93% (Table 2), thus indicating that the framework was developed according to all three aspects listed above, thus relevant and implementable in Ethiopia.

#### Domain 5: Applicability of the framework

Domain 5 of the assessment was concerned with the likely barriers and facilitators to the implementation, strategies to improve uptake, and resource implications of applying the developed framework. The applicability of the framework developed by Chelagat for the improvement of postnatal care in Kenya was assessed in this study specifically focusing on the (1) description of the facilitators and barriers to the application of the framework, (2) provision of advice or tools on how the framework is put into practice, (3) consideration of the potential resources identified in applying the recommendations, and (4) presentation of monitoring and auditing criteria for the implementation of the framework into practice.

The average scaled domain score of domain 5 was 93% (Table 2), thus indicating that the framework was developed according to all aspects listed above. The results from this domain indicate that Chelagat's framework is highly applicable and can easily be implemented to improve postnatal care within the Ethiopian context (Table 2).

#### Domain 6: Editorial independence

Domain 6 of the assessment for the adaptation and contextualization possibility of the framework was concerned with the formulation of recommendations not being unduly biased with competing interests. The assessment considered whether there were competing interests among experts participating in the development of Chelagat's framework. The average scaled score of domain 6 was 94% (Table 2).

This is an indication that the experts who were involved in the development of Chelagat's framework independently participated in the study and contributed their professional expertise to the development thereof, thus adaptable for implementation in Ethiopia.

### Overall assessment of the framework for implementation

The section in the AGREE II, addressed the overall assessment, thus the rating of the overall quality of the framework. It assessed whether the framework developed by Chelagat for the improvement of postnatal care in Kenya could be recommended for use in Ethiopian practice to facilitate improvement of postnatal care (31).

The expectation is that a standardized framework must be based on the scientific evidence and reliable information; it must follow an unbiased process in development, and primarily target the health needs of the public including that of postnatal mothers (38).

The average scaled score of the assessment on this domain was 92% (Table 2). This domain assessed the postnatal care providers' and coordinators' recommendations on the implementation of the framework developed by Chelagat for the improvement of postnatal care within the Kenyan context. The average domain score of 92% was indicative that the framework developed by Chelagat is recommended to be adapted to improve postnatal care in the Ethiopian context.

### The contextualized framework

To contextualize the framework found to be adaptable for the Ethiopian context, the ADAPTE—an international collaboration of guideline developers, researchers, and clinicians who aim to promote the development and use of clinical practice guidelines through the adaptation of existing guidelines—as described by Harrison, Legare, Graham and Fervers (39), was utilized. Factors specific to Ethiopia, such as the health policy, cultural issues, health system, maternal health care utilization, the budget for health care, and available human resources, were taken into consideration in contextualizing the framework for the Ethiopian context. There was no significant difference in the related factors between Kenya and Ethiopia, however, the few aspects specific to Ethiopia, identified by the literature review and the data gathered from respondents, were included as context points in the contextualized framework.

**Figure 2:**
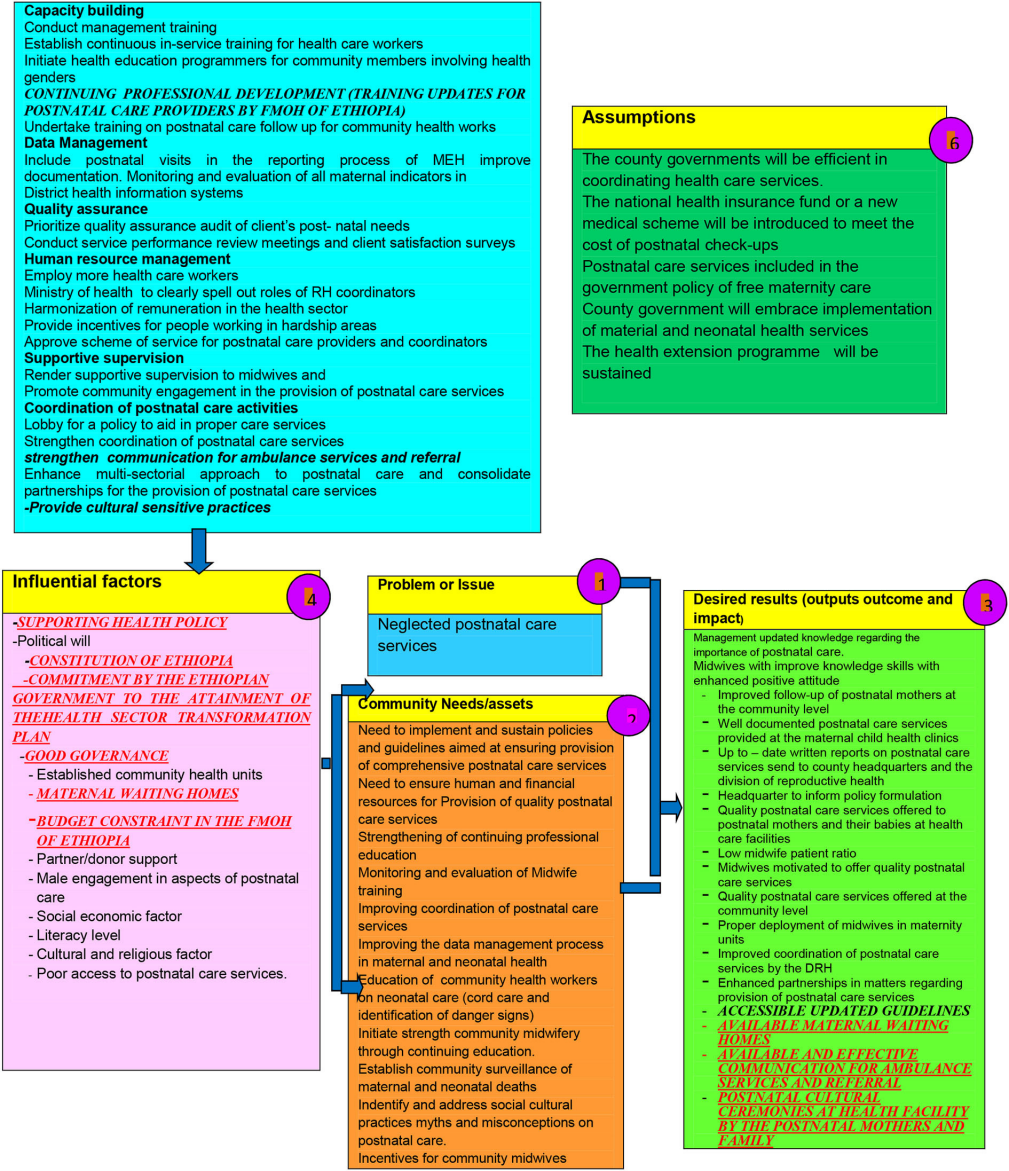
The contextualized framework.

The context points that were identified by the participants and the available literature are indicated in red, bold, underlined, and caps in the contextualized framework. Therefore, as illustrated in Figure 2, the original framework remained as developed and designed by Chelagat (33), and the context points were included to indicate an added information for the contextualized framework for the Ethiopian context.

The following adaptations were included: (1) *Strategies* continuing professional development (training updates for postnatal care providers by FMOH of Ethiopia); (2) *Influential factors* supporting health policy, Constitution of Ethiopia, commitment by the Ethiopian government to the attainment of the health sector transformation plan, good governance, improved maternal waiting homes and budget constraint in the FMOH of Ethiopia; and (3) *Outputs* that include accessible updated guidelines, available maternal waiting homes, available and effective communication with ambulance services, as well as available postnatal cultural ceremonies at a health facility. These adaptations were identified from respondents' recommendations, as well as from the available literature.

## Limitations

There might have been differences in views and opinions among postnatal care providers in unselected health facilities due to possible differences in access to resources for postnatal care.

## Conclusion

The framework developed by Chelagat (2015) to improve postnatal care in Kenya was found suitable for adaptation in the Ethiopian context. With the support of all stakeholders, the framework was adapted to be context specific, thus successfully contextualized for utilization in Ethiopia. Due to the active involvement of all stakeholders during the process, this contextualized framework is owned by postnatal care providers in Ethiopia, positively impacting on the possible success of implementation. However, any framework will only be effective, provided that the methods and strategies used in the framework are shared among the relevant stakeholders responsible for the implementation.

Researchers and policy makers need to utilize all avenues to adapt and contextualize existing frameworks for possible implementation in different contexts.

## Data availability statement

The raw data supporting the conclusions of this article will be made available by the authors, without undue reservation.

## Ethics statement

The study involving human participants were reviewed and approved by the IRB (Ethics Committee) of the University of South Africa. The participants provided their written informed consent to participate in this study.

## Author contributions

Both authors equally contributed to the conceptualization, design, acquisition of data, analysis, interpretation of the data, and drafting of the manuscript. Both authors contributed to the article and approved the submitted version.

## Conflict of interest

The authors declare that the research was conducted in the absence of any commercial or financial relationships that could be construed as a potential conflict of interest.

## Publisher's note

All claims expressed in this article are solely those of the authors and do not necessarily represent those of their affiliated organizations, or those of the publisher, the editors and the reviewers. Any product that may be evaluated in this article, or claim that may be made by its manufacturer, is not guaranteed or endorsed by the publisher.
